# Lack of Effect of Omecamtiv Mecarbil on Smooth Muscle Myosin Phosphatase Target Subunit-1 (MYPT1) Phosphorylation and Hemodynamics in Rats

**DOI:** 10.7759/cureus.87854

**Published:** 2025-07-13

**Authors:** Marina Takata, Mika Nishikawa, Hisaki Hayashi, Aya Yamamura, Keita Saku, Toru Kawada, Motohiko Sato, Shinji Kawahito, Hiroyuki Kinoshita

**Affiliations:** 1 Department of Dental Anesthesiology, Tokushima University Hospital, Tokushima, JPN; 2 Department of Physiology, Aichi Medical University, Nagakute, JPN; 3 Department of Cardiovascular Dynamics, National Cerebral and Cardiovascular Center, Osaka, JPN; 4 Department of Anesthesiology, Seirei Mikatahara General Hospital, Hamamatsu, JPN

**Keywords:** blood pressure, blood vessel, contraction, myosin, omecamtiv mecarbil, vascular smooth muscle

## Abstract

Background

It remains unclear whether omecamtiv mecarbil, a cardiac myosin direct activator, enhances vascular smooth muscle myosin function at clinical and supra-clinical doses.

Aims

The current study evaluated the effect of omecamtiv mecarbil, a cardiac myosin activator, on vascular smooth muscle contraction mediated by myosin phosphatase target subunit-1 (MYPT1) phosphorylation in rats.

Methods

Endothelium-denuded rat aortic rings underwent isometric force recordings (n = 7-9) and western immunoblotting (n = 5) to assess vascular smooth muscle MYPT1 phosphorylation. Aortic rings were incubated with phenylephrine, omecamtiv mecarbil (10^-6^ or 10^-5^ mol/L), or both. Mean arterial pressure and heart rate in rats were measured with (n = 5) or without (n = 5) intravenous administration of omecamtiv mecarbil (10^-5^ mol/L) under general anesthesia.

Results

The clinical (10^-6^ mol/L) and supra-clinical (10^-5^ mol/L) doses of omecamtiv mecarbil did not alter the phenylephrine-induced concentration-response curves (10^-9^ to 10^-5^ mol/L). Omecamtiv mecarbil (10^-5^ mol/L) did not affect vascular smooth muscle MYPT1 phosphorylation induced by phenylephrine (10^-5^ mol/L). Intravenous omecamtiv mecarbil (10^-5^ mol/L) did not change either mean arterial pressure or heart rate under general anesthesia.

Conclusion

At clinical and supra-clinical doses, omecamtiv mecarbil did not alter phenylephrine-induced vascular smooth muscle contraction via MYPT1 phosphorylation or hemodynamic parameters under anesthesia in rats. These findings suggest that omecamtiv mecarbil, even at high doses, does not appear to influence vascular smooth muscle function, supporting its preclinical evidence of vascular safety as a cardiac myosin direct activator.

## Introduction

Omecamtiv mecarbil, a myosin direct activator, binds to the cardiac myosin catalytic domain, priming cardiac myosin for the onset of contraction [1,2]. The agent increases the number of myosin heads available to bind to the cardiac actin filament, facilitating the start of the cardiac cycle [2]. Thus, targeting cardiac myosin may offer a therapeutic approach to systolic heart failure by increasing cardiac contractility. Indeed, omecamtiv mecarbil improves cardiac function in patients with heart failure resulting from reduced left ventricular ejection fraction [3]. Patients treated with omecamtiv mecarbil appear to experience a lower incidence of a composite outcome of heart failure events or death from cardiovascular derangement [3]. Omecamtiv mecarbil significantly improved clinical outcomes in patients with heart failure with reduced ejection fraction and systolic blood pressure of lower than 100 mmHg, predominantly through a reduction in heart failure events, independent of blood pressure changes [4]. However, it remains unclear whether omecamtiv mecarbil, at clinical and supra-clinical doses, affects vascular smooth muscle myosin function, potentially affecting hemodynamic parameters such as blood pressure.

Myosin phosphatase target subunit-1 (MYPT1) is a subunit of myosin phosphatase, and its phosphorylation inhibits myosin phosphatase, leading to vascular smooth muscle myosin activation [5,6]. Indeed, MYPT1 plays a major role in the extent of smooth muscle contraction caused by many vasoactive agents, including phenylephrine [7]. However, it remains unclear whether omecamtiv mecarbil affects vascular smooth muscle contraction mediated by MYPT1 phosphorylation and whether systemic intravenous administration of the agent alters hemodynamics.

The current study evaluated whether omecamtiv mecarbil, a cardiac myosin activator, at clinical and supra-clinical doses, affects vascular smooth muscle contraction mediated by MYPT1 phosphorylation in rats. We conducted ex vivo and in vivo experiments in rat models to assess the effects of clinically relevant omecamtiv mecarbil on vascular smooth muscle function.

## Materials and methods

The study was conducted at three locations: the Department of Dental Anesthesiology, Tokushima University Graduate School of Biomedical Sciences, Tokushima, Japan; the Department of Physiology, Aichi Medical University School of Medicine, Aichi, Japan; and the Department of Cardiovascular Dynamics, National Cerebral and Cardiovascular Center, Osaka, Japan. The ex vivo and in vivo protocols of this study were approved by the Animal Experimentation Committee of Tokushima University (Tokushima, Japan; T2022-98) and the Animal Subject Committee of the National Cerebral and Cardiovascular Center (Osaka, Japan; No.24057), respectively. This study was conducted according to Animal Research: Reporting of In Vivo Experiments (ARRIVE) guidelines [8]. Twelve-week-old male Wistar rats, provided by Japan SLC Inc. (Hamamatsu, Japan), were housed under a 12-hour light-dark cycle (lights on from 08:00 to 20:00) with the room temperature maintained at 21°C until the experiments. The concentrations of drugs used in this study are expressed as final molar concentrations.

Rat aorta preparation

Thoracic aortas were excised from 14 rats anesthetized with 3% isoflurane inhalation in 2 L/min of 100% oxygen and used for organ chamber experiments (n = 9) and western immunoblotting analysis (n = 5). Endothelium-denuded rat aortas were examined in Krebs-Ringer bicarbonate solution (control Krebs-Ringer solution, pH 7.4) bubbled with a 95% O_2_-5% CO_2_ gas mixture. The solution had the following composition (mmol/L): sodium chloride (NaCl) 119, potassium chloride (KCl) 4.7, calcium chloride (CaCl_2_) 2.5, magnesium sulfate (MgSO_4_) 1.17, potassium dihydrogen phosphate (KH_2_PO_4_) 1.18, sodium bicarbonate (NaHCO_3_) 25, and glucose 5.5 [7,9]. Endothelial cells were removed from the aortic rings by rubbing the lumen with a needle to prevent interference from endothelium-derived vasodilator substances [7].

Organ chamber experiments

Endothelium-denuded aortic rings (3 mm in length) were suspended in an organ chamber containing 10 mL of control Krebs-Ringer solution (37°C) bubbled with a 95% O_2_-5% CO_2_ gas mixture. Each ring was connected to an isometric force transducer and stretched to an optimal resting tension of approximately 1.5 g, as determined by the contraction to the α-adrenoceptor agonist phenylephrine (3×10^-7^ mol/L, Sigma-Aldrich LLC, Tokyo, Japan). The absence of relaxation in response to acetylcholine (10^-6^ mol/L) was confirmed to verify endothelial cell removal in each ring [7]. Concentration-response curves for phenylephrine (10^-9^ to 10^-5^ mol/L) were obtained cumulatively at four- to six-minute intervals. The contraction in response to each concentration of phenylephrine was expressed as a percentage of the contraction in response to phenylephrine (3×10^-7^ mol/L) [7]. Some rings were incubated for 15 minutes with omecamtiv mecarbil (CK-1827452, MedChemExpress, Monmouth Junction, USA, 10^-5^ and 10^-6^ mol/L, n = 7 each), whereas control rings received no additional compound (n = 9). The organ bath results were consistent across the rats used and the experimental repeats.

Western immunoblotting analysis

Endothelium-denuded rat aortas were incubated in 10 mL of control Krebs-Ringer solution (37°C) bubbled with a 95% O_2_-5% CO_2_ gas mixture for 15 minutes with or without omecamtiv mecarbil (10^-5^ mol/L). Subsequently, all rings were exposed to phenylephrine (10^-5^ mol/L) for five minutes. We determined the above incubation times based on the incubation time of omecamtiv mecarbil (10^-5^ mol/L) and the duration of phenylephrine (10^-5^ mol/L)-induced contraction in the organ chamber experiments. All aortic rings were frozen and stored at -80°C until use. The protein fraction was extracted from the aortic ring homogenates using T-PER buffer (Thermo Fisher Scientific K.K., Tokyo, Japan). Extracted protein (30 µg/lane) was separated on an 8% acrylamide gel and then transferred to an Immobilon-P PVDF membrane (Sigma-Aldrich LLC, Tokyo, Japan). The membrane was blocked with Tris-buffered saline containing 5% bovine serum albumin (Sigma-Aldrich LLC, Tokyo, Japan) and 0.1% Tween 20 (Sigma-Aldrich LLC, Tokyo, Japan) at room temperature for three hours and then incubated with a primary antibody (1:500-800) specific for either phospho-MYPT1 (p-MYPT1, #5163, Cell Signaling Technology Japan, K.K., Tokyo, Japan; sc-377531, Santa Cruz Biotechnology, Inc., Dallas, TX, USA) or total-MYPT1 (t-MYPT, sc-514261, Santa Cruz Biotechnology, Inc., Dallas, TX, USA) at 4°C for 18 hours. After three washes in Tris-buffered saline containing 0.1% Tween 20, immunoblotted membranes were treated with an anti-rabbit or anti-mouse horseradish peroxidase-conjugated IgG secondary antibody (1:5000; #170-6515, #170-6516, Bio-Rad Laboratories, Hercules, CA, USA) at room temperature for one hour. After three additional washes in Tris-buffered saline containing 0.1% Tween 20, blotting signals were detected using an ImmunoStar LD reagent (Fujifilm Wako Pure Chemical, Osaka, Japan), and images were analyzed using the Imager 600 system (GE HealthCare Technologies, Chicago, IL, USA). Protein expression levels were normalized using anti-glyceraldehyde-3-phosphate dehydrogenase (GAPDH, 1:5000; GT239, GeneTex, Funakoshi Co., Ltd., Tokyo, Japan) and anti-mouse horseradish peroxidase-conjugated IgG (1:10000) antibodies. We quantified blot band intensities using ImageJ (Ver. 1.54). The blot data were consistent across the rats used and the experimental repeats. Replicate western blots were performed to confirm reproducibility and representative results.

In vivo hemodynamic measurements

Rats (n = 10) were anesthetized with an intraperitoneal injection (2 mL/kg) of a mixture of α-chloralose (40 mg/mL) and urethane (250 mg/mL), followed by continuous intravenous administration of the anesthetic mixture (diluted 18-fold with physiological saline, 2 mL/kg/h) through a polyethylene catheter (PE-50, Becton Dickinson and Company, Sparks, MD, USA) inserted into the left femoral vein. Venous and arterial catheters (PE-50) were inserted into the right femoral vein and right femoral artery, respectively. Arterial blood pressure was measured using a pressure transducer (DX-200, Nihon Kohden, Tokyo, Japan), and a lactate Ringer’s solution was administered (4 mL/kg/h) to maintain fluid balance. Each rat was intubated from the trachea and mechanically ventilated at 80 cycles/min with 50% oxygen, and body temperature was maintained at 37℃ using heating equipment. The heart rate was monitored using a cardiotachometer (AT-601G, Nihon Kohden, Tokyo, Japan). Hemodynamic data were acquired after a stabilization period of at least 30 minutes following surgical preparation. The effects of omecamtiv mecarbil (10^-5^ mol/L, n = 5) or an equivalent volume of the vehicle containing 0.125% dimethyl sulfoxide (time control, n = 5) on hemodynamics were monitored for 30 minutes. We terminated each in vivo study 30 minutes after its start. A rat blood volume of 6 mL per 100 g body weight was used to calculate the administered doses [7,10]. At the end of the experiment, the rats were euthanized under deep anesthesia.

Statistical analysis

Statistical analysis was performed using Predictive Analytics SoftWare (PASW) Statistics 18 (IBM Japan Inc., Tokyo, Japan). Variables are expressed as mean ± SD, and data were analyzed using paired t-tests or one-way ANOVA with Scheffe’s test. Differences were considered statistically significant at P < 0.05. Sample size estimation was based on previous studies [7,9] without a formal a priori power analysis.

## Results

Organ chamber experiments

**Figure 1:**
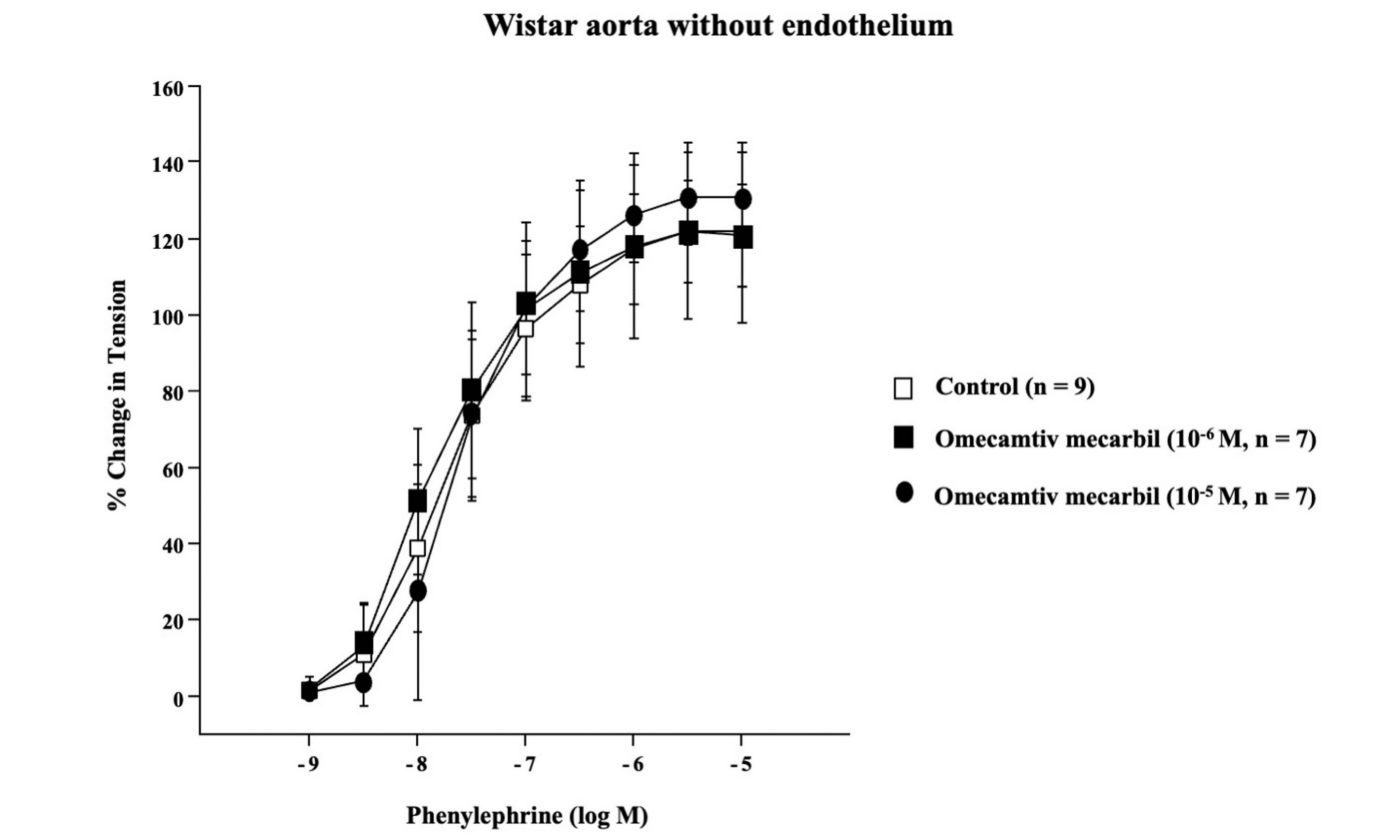
Concentration response curves in response to phenylephrine.

The cumulative addition of phenylephrine (10^-9^ to 10^-5^ mol/L) induced concentration-dependent contractions in endothelium-denuded rat aortas. Omecamtiv mecarbil treatment at 10^-6^ mol/L or 10^-5^ mol/L for 15 minutes did not alter the concentration-response curves for phenylephrine (10^-9^ to 10^-5^ mol/L, Figure 1).

Western immunoblotting analysis

**Figure 2:**
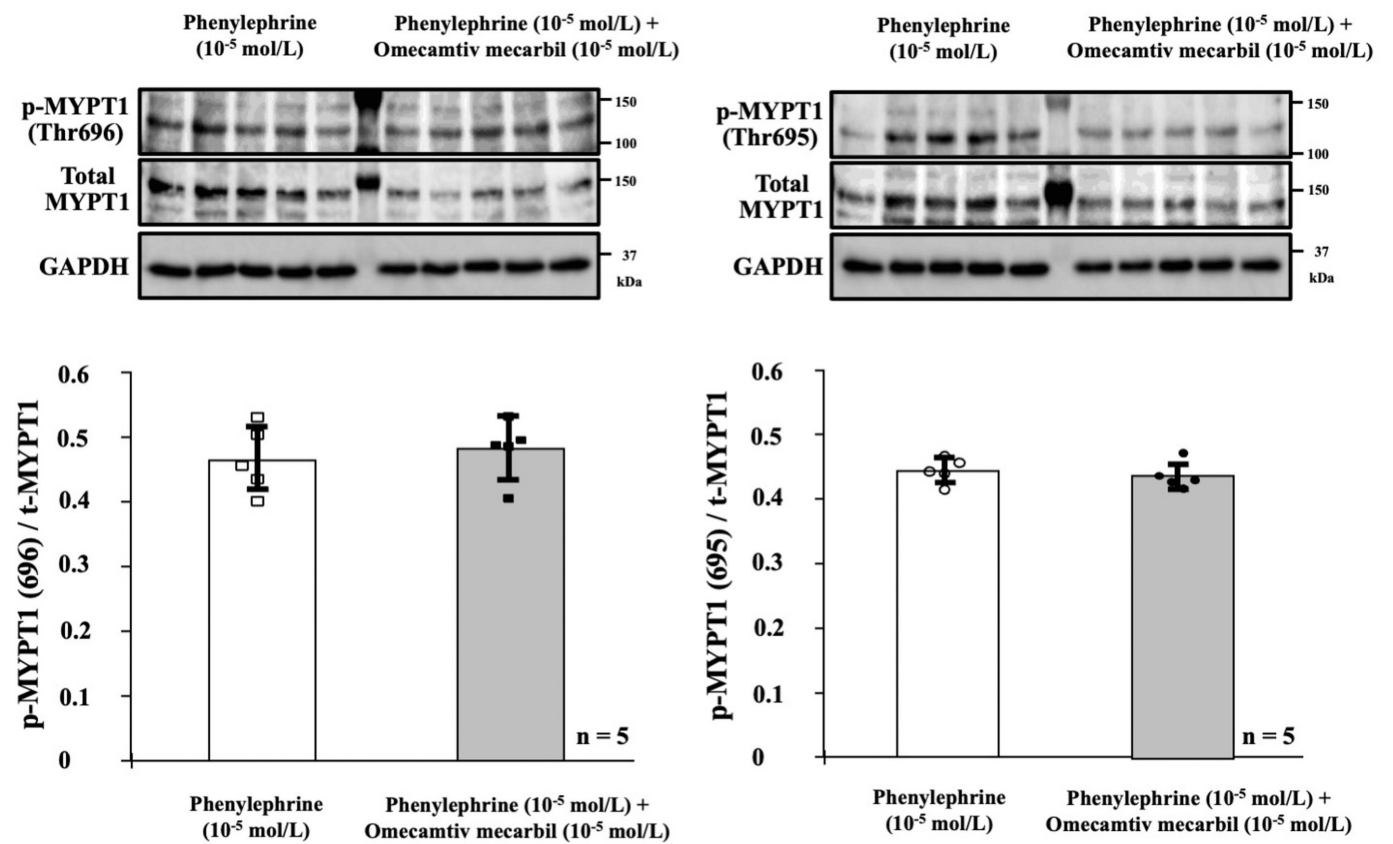
The MYPT1 phosphorylation levels at Thr696 or Thr695 in aortic ring protein fractions. Myosin phosphatase target subunit 1 (MYPT1) phosphorylation at Thr696 or Thr695 was assessed in protein fractions extracted from the aortic rings incubated with phenylephrine (10^-5^ mol/L) alone or in combination with omecamtiv mecarbil (10^-5^ mol/L). The upper two panels show immunoblot signals detected using antibodies targeting p-MYPT (Thr696), p-MYPT (Thr695), t-MYPT, and glyceraldehyde-3-phosphate dehydrogenase (GAPDH). Note that phenylephrine (10^-5^ mol/L) alone and in combination with omecamtiv mecarbil (10^-5^ mol/L) induced similar levels of MYPT1 phosphorylation at both sites.

MYPT1 phosphorylation at Thr696 or Thr695 was evaluated using protein fractions extracted from aortic rings incubated with phenylephrine (10^-5^ mol/L) alone or in combination with omecamtiv mecarbil (10^-5^ mol/L). Phenylephrine (10^-5^ mol/L) alone enhanced MYPT1 phosphorylation in the aortic rings (Figure 2). However, phenylephrine (10^-5^ mol/L) and phenylephrine (10-5 mol/L) in combination with omecamtiv mecarbil (10^-5^ mol/L) resulted in similar p-MYPT1/t-MYPT values, as detected using two different antibodies specific for p-MYPT1 at Thr696 and Thr695 (Figure 2).

In vivo hemodynamic measurements

**Figure 3:**
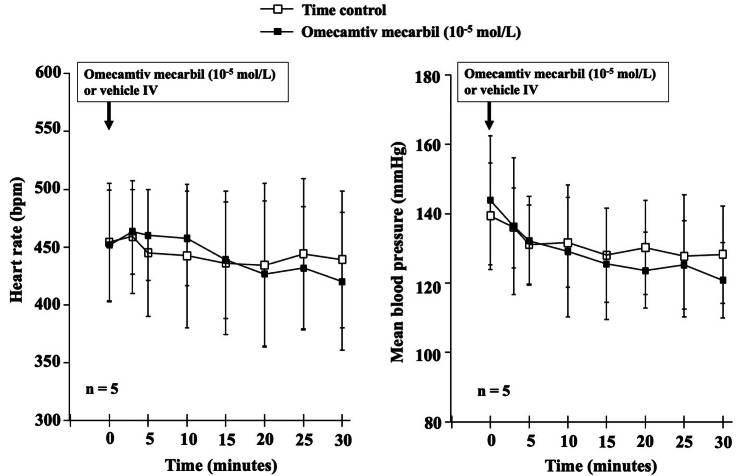
Time course of changes in heart rate and mean arterial pressure following intravenous omecamtiv mecarbil. The time course of heart rate (bpm) and mean arterial pressure (mmHg) is shown following intravenous administration of omecamtiv mecarbil (10^-5^ mol/L) or equivalent volume of vehicle (time control) until a 30-minute period. Note that neither omecamtiv mecarbil nor the vehicle altered mean arterial pressure or heart rate in rats under general anesthesia induced with a mixture of α-chloralose and urethane.

Intravenous omecamtiv mecarbil (10^-5^ mol/L, n = 5) or an equivalent volume of vehicle (time control, n = 5) did not alter mean arterial pressure or heart rate until 30 minutes in rats under general anesthesia with a mixture of α-chloralose and urethane (Figure 3). We did not observe any effects of α-chloralose and urethane anesthesia itself on cardiovascular parameters (data not shown).

## Discussion

Malik et al. [1] first reported in 2011 that omecamtiv mecarbil, a cardiac myosin direct activator, binds to the myosin catalytic domain and increases the transition rate of myosin into the force-generating actin-bound state, supporting continuous cardiac muscle contraction [1]. Subsequent studies have shown that omecamtiv mecarbil stabilizes an elongated distal myosin domain and primes cardiac myosin for the onset of contraction [2]. These findings led clinicians to hypothesize that a direct activator of cardiac myosin could serve as a treatment for systolic heart failure. Supporting this, the first-in-human data published by Teerlink et al. [11] demonstrated a dose-dependent enhancement in left ventricular systolic function with omecamtiv mecarbil administration. More recent research has further confirmed improved cardiac function in patients with heart failure and reduced left ventricular ejection fraction [3]. In this study, treatment with the myosin direct activator was associated with a lower incidence of a composite outcome of heart failure events or cardiovascular death compared to placebo, over a median follow-up of 21.8 months [3]. Notably, omecamtiv mecarbil also improved clinical outcomes in patients with heart failure with reduced ejection fraction and low systolic blood pressure (≦ 100 mmHg), predominantly by reducing heart failure events independently of blood pressure changes [4]. Nevertheless, it remains unclear whether omecamtiv mecarbil also influences vascular smooth muscle myosin function, potentially affecting hemodynamic parameters such as blood pressure.

Previous studies have shown that therapeutic plasma concentrations of omecamtiv mecarbil range from 100 to 300 ng/ml, corresponding to clinical concentrations below 10^-6^ mol/L [12,13]. In the current study, treatment with omecamtiv mecarbil at 10^-6^ mol/L or 10^-5^ mol/L for 15 minutes did not alter the concentration-response curves to phenylephrine (10^-9^ to 10^-5^ mol/L) in the endothelium-denuded aortic rings. Therefore, our results suggest that both clinical and supra-clinical concentrations of omecamtiv mecarbil do not affect α1-adrenoceptor agonist-induced, endothelium-independent contraction in rats. However, the absence of the contractile effects by omecamtiv mecarbil could be due to limitations in sensitivity, exposure time, or tissue specificity. Additionally, we cannot rule out the possibility that omecamtiv mecarbil may influence vascular tone indirectly through neurohormonal mechanisms or metabolic effects.

In the present study, a high concentration of phenylephrine alone enhanced MYPT1 phosphorylation in endothelium-denuded aortic rings. However, phenylephrine in combination with a supra-clinical concentration of omecamtiv mecarbil did not further increase p-MYPT1/t-MYPT values, which were detected using two different antibodies targeting p-MYPT1 at Thr696 and Thr695. MYPT1 is one of the subunits of myosin phosphatase, and MYPT1 phosphorylation leads to inhibition of myosin phosphatase activity, thereby playing a significant role in vascular smooth muscle myosin activation [5,6]. Indeed, MYPT1 is an important determinant of the extent of smooth muscle contraction induced by various vasoactive agents, including phenylephrine [7]. Therefore, these findings support the conclusion that both clinical and supra-clinical doses of omecamtiv mecarbil do not affect MYPT1 phosphorylation-mediated vascular smooth muscle myosin activation.

We observed that supra-clinical doses of intravenous omecamtiv mecarbil did not alter hemodynamic parameters, including mean arterial pressure and heart rate, under general anesthesia. We chose supra-clinical concentrations in the in vivo study since we noted that treatment with omecamtiv mecarbil at 10^-5^ mol/L did not alter the concentration-response curves to phenylephrine. However, it is still unclear how the clinical and supra-clinical concentrations of omecamtiv mecarbil relate to safety margins or potential overdose scenarios in clinical settings. In addition, general anesthesia might have masked subtle hemodynamic effects, suggesting that future studies using conscious models would be beneficial.

We must note several limitations of the current study. First, we did not examine the effects of omecamtiv mecarbil on vascular smooth muscle in arteries from different organs, including the coronary artery. Therefore, we cannot conclude the role of omecamtiv mecarbil at clinical and supra-clinical concentrations in the regulation of regional circulation. However, the lack of hemodynamic changes observed in the in vivo model suggests that the circulatory effects of the supra-clinical concentration tested are likely negligible. Second, we did not verify the impact of the supra-clinical concentration of omecamtiv mecarbil on myosin light chain phosphorylation, as we could not identify an appropriate antibody for detecting myosin light chain in rat vascular smooth muscle. Therefore, this is an area that needs further confirmation. Third, our findings are based on a single vascular bed (the aorta) and are obtained under anesthesia, which limits their generalizability to all vascular tissues or conscious states. Fourth, the current study lacks a power analysis, and it was conducted without randomization and blinding. However, sample size estimation was based on previous studies [7,9] without a formal a priori power analysis. Fifth, the absence of significant changes caused by omecamtiv mecarbil might be due to potential limitations in sensitivity, sample size, or assay resolution. Further studies, including those in other vascular tissues, chronic exposure models, or human-relevant systems, will be needed to address these limitations.

## Conclusions

Both clinical and supra-clinical doses of omecamtiv mecarbil did not affect phenylephrine-induced contraction, MYPT1 phosphorylation in vascular smooth muscle, or hemodynamic parameters under anesthesia in rats. Even at high doses, omecamtiv mecarbil does not appear to influence vascular smooth muscle function, supporting its preclinical evidence of vascular safety as a cardiac myosin direct activator.
